# Determinants and Temporal Trends of Perfluoroalkyl Substances in Pregnant Women: The Hokkaido Study on Environment and Children’s Health

**DOI:** 10.3390/ijerph15050989

**Published:** 2018-05-14

**Authors:** Meng-Shan Tsai, Chihiro Miyashita, Atsuko Araki, Sachiko Itoh, Yu Ait Bamai, Houman Goudarzi, Emiko Okada, Ikuko Kashino, Hideyuki Matsuura, Reiko Kishi

**Affiliations:** 1 Center for Environmental and Health Sciences, Hokkaido University, Sapporo 060-0812, Japan; ms.tsai1006@gmail.com (M.-S.T.); miyasita@med.hokudai.ac.jp (C.M.); AAraki@cehs.hokudai.ac.jp (A.A.); vzbghjn@den.hokudai.ac.jp (S.I.); u-aitbamai@med.hokudai.ac.jp (Y.A.B.); ghouman@cehs.hokudai.ac.jp (H.G.); 2 Graduate School of Health Sciences, Hokkaido University, Sapporo 060-0812, Japan; 3 Institute of Occupational Medicine and Industrial Hygiene, National Taiwan University, Taipei 10055, Taiwan; 4 Division of Respiratory Medicine, Graduate School of Medicine, Hokkaido University, Sapporo 060-8638, Taiwan; 5 Department of Public Health, Graduate School of Medicine, Hokkaido University, Sapporo 060-8638, Japan; ekat_oka@yahoo.co.jp (E.O.); ikukomax2007@yahoo.co.jp (I.K.); 6 Laboratory of Bioorganic Chemistry, Division of Applied Bioscience, Research Faculty of Agriculture, Hokkaido University, Sapporo 060-8589, Japan; matsuura@chem.agr.hokudai.ac.jp

**Keywords:** perfluoroalkyl substances, temporal trends, pregnant women

## Abstract

Perfluoroalkyl substances (PFAS) are persistent bio-accumulative chemicals that impact the health of pregnant women and their children. PFAS derive from environmental and consumer products, which depend on human lifestyle, socioeconomic characteristics, and time variation. Here, we aimed to explore the temporal trends of PFAS in pregnant women and the characteristics related to maternal PFAS concentration. Our study is part of the Hokkaido Study on Environment and Children’s Health, the Hokkaido large-scale cohort that recruited pregnant women between 2003 and 2011. Blood samples were acquired from pregnant women during the third trimester to measure PFAS and cotinine concentrations. Maternal basic information was collected with a baseline structured questionnaire. Eleven PFAS were measured from 2123 samples with ultra-performance liquid chromatography coupled with triple quadrupole tandem mass spectrometry. Eight PFAS were above 80% detection rate and were included in the final analysis. We used multivariable linear regression to analyze the association of pregnant women characteristics with the levels of eight PFAS. The temporal trend of PFAS was observed in two periods (August 2003 to January 2006 and February 2006 to July 2012). The concentration of perfluorooctane sulfonate (PFOS) significantly decreased from August 2003 to January 2006 and from February 2006 to July 2012. The concentrations of perfluorododecanoic acid (PFDoDA), perfluoroundecanoic acid (PFUnDA), and perfluorotridecanoic acid (PFTrDA) increased significantly between August 2003 and January 2006, whereas they decreased significantly between February 2006 and July 2012. Women with pre-pregnancy body mass index (BMI) >25 kg/m^2^ had lower PFUnDA, PFDoDA, and PFTrDA levels than did those with normal BMI (18.5–24.9 kg/m^2^). Pregnant women, who were active smokers (cotinine > 11.49 ng/mL), had higher PFOS than the non-smokers (cotinine < 0.22 ng/mL). Lower levels of PFHxS, PFOS, PFOA, PFNA, and PFDA were observed in women, who had given birth to more than one child. There were also significant positive associations between PFAS levels and annual income or maternal education. PFAS levels varied in women with higher pre-pregnancy BMI, active smoking status, higher education level and annual income. The causes of the individual PFAS differences should be explored in an independent study.

## 1. Introduction

Since the 1950s, perfluoroalkyl substances (PFAS) have been widely prevalent in the world. PFAS are man-made substances, identified as endocrine disruptor chemicals and persistent organic pollutants, which may exist in the environment for long periods [1]. Perfluorooctanoic acid (PFOA) and perfluorooctane sulfonate (PFOS) was firstly introduced for manufacture, use, and consumer products such as cement, fire-fighting formulations, and varnishes etc., and also produced larger emission in the environment [2]. International organizations, such as the United States Environmental Protection Agency (U.S EPA) and the Registration, Evaluation, Authorization and restriction of Chemicals (REACH) have noted the impact of PFOA and PFOS in humans and the environment since 2002 [3,4]. In 2006, U.S EPA invited the main PFAS producers to join the PFOA stewardship program to eliminate PFOA and related chemicals. However, long- (>8 carbons) and short- (<8 carbons) chain PFAS have replaced PFOS and PFOA [5]. Some studies reported that PFOS and PFOA concentrations have decreased due to regulation [6,7]. In addition to PFOS and PFOA, studies focusing on other long- and short-chain PFAS produced inconsistent results and are limited in number [8,9]. One study investigating PFOS and PFOA concentrations in the surface water of Japan found that PFOS and PFOA existed in the surface water of Japan [10]. The highest PFOS and perfluorohexane sulfonate (PFHxS) concentrations were in Kinki area; the lowest were in Hokkaido and Tohoku areas [11]. Due to their long half-life, PFAS may persist in the environment for long time, independently of their concentration. According to our previous studies, PFAS showed a significant impact on infant, children, and maternal health, including neurodevelopment [12], thyroid function [13], reproductive [14] and steroid hormones [15], adipokines [16], asthma and allergies [17,18], and maternal fatty acids [19].

Infant and children health is related to maternal characteristics such as maternal age and lifestyle during or before pregnancy. People may be exposed to PFAS in their living environment, through contaminated food, food package, and drinking water [20,21]. However, only a few studies investigated the characteristics of pregnant women, including maternal delivery age [22], body mass index (BMI) [23], education level [24], smoking status [25], and household income [26], which were all in relation to different carbon chain of PFAS levels, especially to PFOA and PFOS; however, their results were inconsistent. In addition, most studies reported temporal trend of PFAS with various sampling time and different trend for each PFAS except PFOA and PFOS [6,7,27,28]. We previously reported the association between PFAS levels in pregnant women and the temporal trend of PFASs, based on a small sample size [29]. However, there is no study investigating the association between PFAS levels and maternal characteristics during pregnancy; in addition, the temporal trend of PFAS due to regulation, based on large cohort birth study, has not been described clearly in Asia. In this study, we aimed to explore the temporal trend of PFAS in pregnant women and the characteristics related to different maternal PFAS concentrations.

## 2. Materials and Methods

### 2.1. Study Populat

Study participants included pregnant women, based on a prospective birth cohort (the Hokkaido Study on Environment and Children’s Health; the Hokkaido large-scale cohort) from February 2003 through March 2012, and details have been described previously [30,31,32]. Briefly, eligible women during early pregnancy (<13 weeks gestational age) visited the hospitals and were introduced in the study in Hokkaido area. A total of 20,926 women registered to participate in the study with a written informed consent. Details of the selected PFAS to be measured were described independently [17]. Briefly, we obtained a set of 15,703 participants’ baseline questionnaires, third trimester blood samples, and the birth records of their newborns. Of these, 10,102 participants completed the follow-up questionnaire at four months, which was confirmed to the baseline information in the study. Of these, 2138 pregnant women provided a sufficient number of blood samples during the third trimester to measure PFAS and cotinine levels. We excluded individuals with potentially biased PFAS and/or cotinine concentrations, e.g., those that provided blood samples <26 weeks gestational age (*n* = 15). Finally, a total of 2123 individuals were included in the study. The study protocol was approved by the ethics review board for epidemiological studies at Hokkaido University Graduate School of Medicine (31 March 2003) and the Hokkaido University Center for Environmental and Health Sciences (Reference No. 14, 22 March 2012).

### 2.2. Measurement of PFAS and Cotinine Concentrations in Maternal Blood

A 10 mL blood sample at the third trimester was collected from each pregnant women and directly stored at −80 °C before analysis. Detailed procedures for PFAS analysis have been described previously [29]. Briefly, maternal plasma was analyzed by ultra-performance liquid chromatography coupled with triple quadrupole tandem mass spectrometry instrumentation (Waters Co., Midfield, MA, USA) after basic sample preparation. The concentration of eleven PFASs in 2138 maternal plasma samples was measured, including perfluorohexane sulfonate (PFHxS), perfluorohexanoic acid (PFHxA), perfluoroheptanoic acid (PFHpA), PFOS, PFOA, perfluorononanoic acid (PFNA), perfluorodecanoic acid (PFDA), perfluoroundecanoic acid (PFUnDA), perfluorododecanoic acid (PFDoDA), perfluorotridecanoic acid (PFTrDA), and perfluorotetradecanoic acid (PFTeDA). The method detection limit (MDLs) for PFHxA, PFHpA, PFDA, PFUnDA, PFDoDA, PFTrDA, and PFTeDA was 0.1 ng/mL, for PFOA and PFHxS was 0.2 ng/mL, and for PFNA and PFOS was 0.3 ng/mL. Detailed information regarding blood cotinine measurement was described previously [33]. Maternal cotinine levels were analyzed using the highly sensitive enzyme-linked immunosorbent assay (ELISA) (Cosmic Corporation, Tokyo, Japan). The detection limit of plasma cotinine was 0.12 ng/mL.

### 2.3. Statistical Analysis

As PFAS and cotinine levels were not normally distributed, we converted our data to natural log scale. Because some PFAS concentrations were below methods detection limit (MDL), we assigned a value equal to half MDL in the analysis. Detection rates of PFHxA, PFHpA, and PFTeDA were below 50%, thus these PFASs were excluded from the final analysis. Similarly, we assigned a value of half MDL for cotinine, when participants’ cotinine levels were below MDL. Regarding the related variables for maternal characteristics, age at delivery (<25 years old, 25–29 years old, 30–34 years old, and ≥35 years old) and education level (≤9 years, 10–12 years, 13–16 years, and ≥17 years) were divided into four categories, pre-pregnancy BMI (<18.5 kg/m^2^, 18.5–24.9 kg/m^2^, and ≥25 kg/m^2^), parity (0, 1, and ≥2), and smoking habits in three (non-smokers, cotinine <0.22 ng/mL; passive smokers cotinine 0.22–11.49 ng/mL and active smokers, cotinine >11.49), according to a previous publication (Sasaki et al., 2011), and alcohol consumption in two (never alcohol drinking or with alcohol drinking history). In addition, the annual household income (<3 million yen, 3–5 million yen, and >5 million yen) was included in the analysis. Linear regression was used to analyze the temporal trends between 2003 and 2012 in 2123 pregnant women, according to the time of blood acquisition. We recruited pregnant women at their first trimester (2003–2011) and they provided blood at their third trimester (2003–2012), therefore we used the blood sampling time to estimate the temporal trend of PFAS in this study. Because the sampling time started on August 2003 and to balance the sample size, we divided the half year based on sampling time as a group. Moreover, U.S EPA launched the PFOA Stewardship Program in January 2006 (2010/2015 PFOA Stewardship Program [3]), thus we used January 2006 as a cut-off point to observe the differences before (August 2003 to January 2006) and after (February 2006 to July 2012) the action. We used multivariable linear regression to analyze and calculate the crude and adjusted values of PFAS levels for each category of the variables compared to the reference group, and we also tested the trend for each group except for the pre-pregnancy BMI. Maternal age at delivery, pre-pregnancy BMI, maternal education, household income, and parity were adjusted in the full model. Potential confounders were selected by literature review and more than 10% change of the estimate in the model. Statistical significance was defined as *p*-value < 0.05. All statistical analyses in the study were performed with SPSS for Windows version 19.0 J (SPSS, Inc., Chicago, IL, USA).

## 3. Results

Maternal basic characteristics are shown in Table 1. Delivery age was between 30 and 34 years old in 41.5% of mothers; the pre-pregnancy BMI was between 18.5 and 24.9 kg/m^2^ in 73.2% of mothers. Approximately 45.3% participants were first-time mothers, 44% received education between 13–16 years and 41.2% between 10–12 years. Based on cotinine levels at the third trimester, mothers who were passive smokers were in 47.9% and non-smokers were in 43.7%. Most mothers (57.1%) had a history of alcohol consumption. The annual household income was between 3 and 5 million Japanese yen in 45.2% of mothers.

Table 2 shows the distribution of the concentration of 11 PFAS. Detection rate of PFHxA, PFHpA, and PFTeDA was under 50%, therefore we finally included eight PFASs with detection rates above 80%, including PFHxS (geometric mean (GM): 0.34 ng/mL)), PFOS (GM: 4.96 ng/mL), PFOA (GM: 2.06 ng/mL), PFNA (GM: 1.19 ng/mL), PFDA (GM: 0.51 ng/mL), PFUnDA (GM: 1.35 ng/mL), PFDoDA (GM: 0.19 ng/mL), and PFTrDA (GM:0.32 ng/mL).

Mean values and 95% confidence intervals for the mean of PFAS for each maternal characteristic are shown in Table 3. Mean values of all PFASs were significantly different for the education level (*p* < 0.05); however, mean values of all PFASs did not vary significantly for alcohol consumption history. Mean values of PFHxS, PFOS, PFOA, PFNA, and PFDA were also different on parity and annual household income (*p* < 0.05).

Crude and adjusted associations between PFAS and maternally related characteristics are showed in Table S1 and Table 4. PFUnDA and PFTrDA concentrations increased with maternal delivery age in different age groups in the crude model, but only PFUnDA showed significant positive associations across different groups in the adjusted model (*p* for trend = 0.025); <25 years old as a reference, 25–29 years old (β = 0.103, 95% CI: (0.015, 0.191)), 30–35 years old (β = 0.118, 95% CI: (0.031, 0.205)), and ≥35 years old (β = 0.131, 95% CI: (0.036, 0.227)). Compared to the reference group (BMI 18.5~24.9), maternal pre-pregnancy BMI <18.5 showed higher PFNA concentration (β = 0.058, 95% CI: (0.006, 0.11)); PFUnDA (β = −0.081, 95% CI: (−0.161, −0.001)), PFDoDA (β = −0.175, 95% CI: (−0.252, −0.097)), and PFTrDA (β = −0.167, 95% CI: (−0.243, −0.092)) concentrations were significantly lower in individuals with pre-pregnancy BMI >25 in the crude model. In the adjusted model, the association between PFUnDA and maternal pre-pregnancy BMI disappeared. Mothers, who had given birth to one or to more than two babies in the past, showed lower levels of PFHxS, PFOS, PFOA, PFNA, and PFDA compared to those that did not (all p for trends <0.001), in both models. There was a significant correlation between the levels of eight PFAS and higher education level, in both models (*p* for trend <0.05). Moreover, we observed more significant association in PFDA and PFUnDA across different groups. PFNA, PFDA, PFUnDA, and PFDoDA levels were lower in mothers who were active smokers in the crude model; PFOA and PFOS levels were higher in mothers who were active smokers in the adjusted model. Mothers with a history of alcohol consumption had significantly lower PFDA concentration compared to those without, in both models. There was a significant positive correlation between PFHxS, PFOS, PFOA, and PFNA with the annual household income in the adjusted model (*p* for trend <0.05).

**Figure 1:**
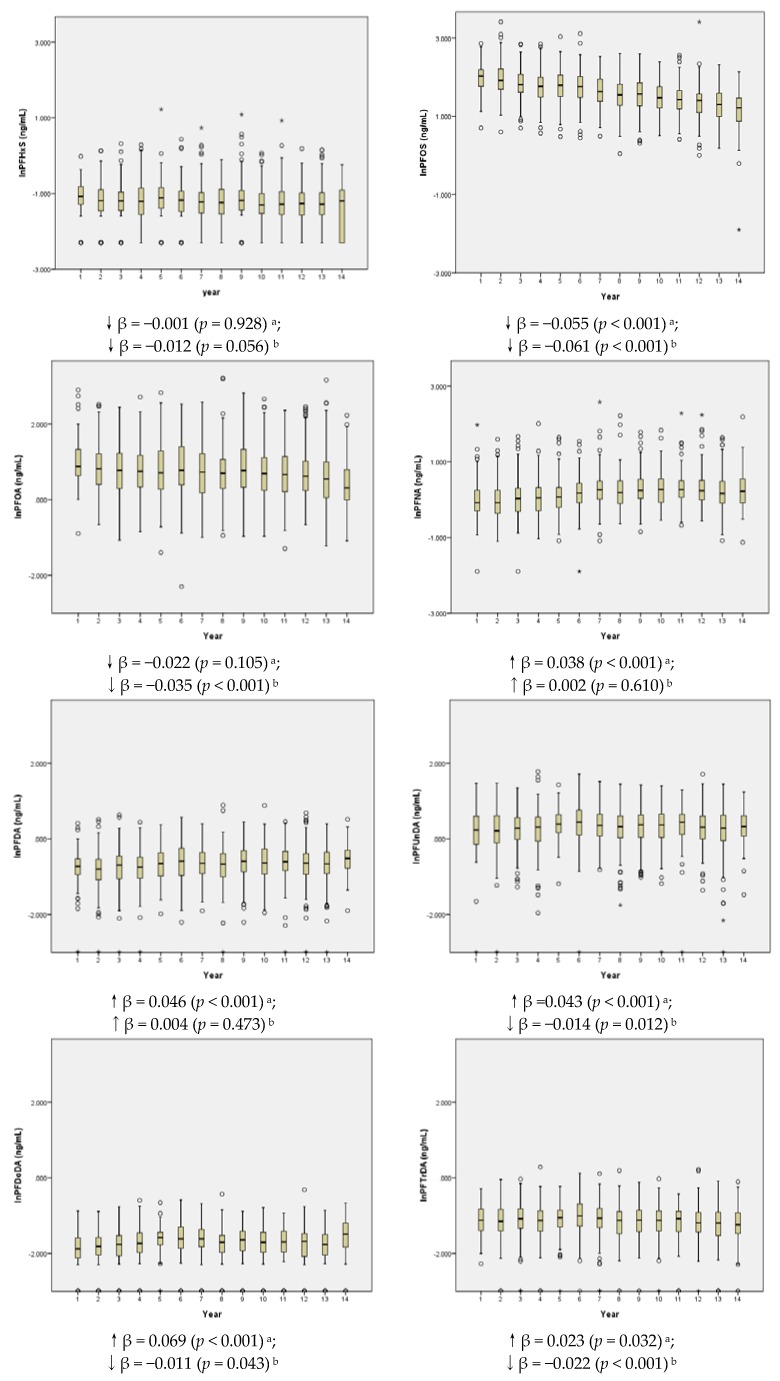
PFAS temporal trend of Hokkaido Study in 2123 pregnant women ^c^. (^a^ represented the period between August 2003 and January 2006; ^b^ represented the period February 2006 and July 2012; ^c^ linear regression adjusted for age and parity; Bold represented *p* value < 0.05; Detail blood collection period and sample size at each period please see the Supplementary Table S2.).

Figure 1 shows the PFAS temporal trends of the Hokkaido Study in 2123 pregnant women from 2003–2012. PFHxS concentration did not change between August 2003 and January 2006 (before the action) and between February 2006 and July 2012 (after the action). PFOS concentration decreased significantly during both periods (*p* < 0.001). PFOA concentration decreased significantly after the action (β = −0.035, *p* < 0.001). PFNA and PFDA concentrations increased significantly before the action (*p* < 0.001), but did not change after the action. PFUnDA, PFDoDA, and PFTrDA concentrations increased significantly before the action, whereas they decreased significantly after the action.

## 4. Discussion

In the present study, we found that women with older delivery age had higher PFUnDA levels; women with pre-pregnancy BMI > 25 had lower PFDoDA and PFTrDA levels; levels of carbon C6 to C10 PFASs were negatively associated with parity; a positive association between maternal education and PFAS levels; higher annual household income was associated with higher PFOS and PFOA levels; passive smokers had higher PFOS and PFOA levels, whereas active smokers had lower PFUnDA; mothers with a history of alcohol consumption had lower PFDA concentration. In addition, PFHxS, PFOS, PFOA, and PFTrDA levels decreased during the period of our study, whereas PFNA, PFDA, and PFDoDA levels increased and PFUnDA level did not change.

### 4.1. Maternal Delivery Age

We found that maternal PFUnDA concentration was positively associated with maternal delivery age, consistent with reported results. Bjerregaard-Olesen et al. recently reported that most PFASs were higher in pregnant women with older age [24]. Wilhelm et al. reported that mother’s age was positively associated with maternal PFOS, PFOA, PFHxS, and PFNA levels [28]. Berg et al. also found that age was positively associated with maternal PFNA, PFDA, and PFUnDA levels [6]. Moreover, Lee et al. found that PFOS levels in the breast milk were also positively associated with age [34]; Lien et al. also reported that cord blood showed a positive association with maternal age from different kinds of specimens [35]. However, Ode et al. found no association between maternal PFAS levels and maternal age [36]. Most studies and the present study reported that mothers with older age had higher PFAS concentration, especially for long-chain PFASs. Currently, PFUnDA has the longest half-life of 9.7 years in humans [8]. Therefore, PFUnDA may accumulate in the body, because the excretion rate may be longer than other PFAS.

### 4.2. Pre-Pregnancy BMI

There was a negative association between PFAS and maternal pre-pregnancy BMI in this study. However, previous studies showed inconsistent results. No association was reported by Kristensen et al. and Ode et al. [9,36]. In contrast, a positive association was found by Fei et al. [23] and Brantsaeter et al. [25]. However, a negative association was reported by Berg et al., Laurintzen et al. and Wilhelm et al. [6,28,37]. In addition, Jensen et al. and Bjerregaard-Olesen found that women with normal BMI had higher PFAS levels [38]. PFASs are mainly distributed to non-fatty organs such as the liver and the kidney, with lower levels accumulating in the adipose tissue [39]. This may explain the lack of positive association between PFAS and maternal pre-pregnancy BMI. Moreover, the main role of the adipose tissue is to store energy in the form of lipids and to regulate lipid profiles. PFAS mainly serve as activators of peroxisome proliferator-activated receptors (PPARs), which are key in lipid metabolism. Animal studies indicated an inverse association between PFAS and lipid levels [40], consistent with our previous study [19]. However, because the body composition is complicated, more studies are required to determine causation.

### 4.3. Parity

We found that compared to the C11-C13, C6-C10 PFAS were significantly negatively associated with parity. According to published papers, most studies found that first pregnancy or nulliparity was associated with higher maternal PFAS levels [6,23,25,26,36,38], cord blood PFAS levels [35] and breast milk PFAS levels [34]. PFAS may be transferred to infants or to the placenta through the umbilical cord [41]. Parity was the largest determinant factor for PFAS concentration, especially parous women had lower PFAS concentration than nulliparous women [25]. Also, blood loss during delivery may decrease maternal body burden of PFAS [42]. On the other hand, because of the shorter half-life of C6-C10 PFAS, they may be easily excreted during delivery.

### 4.4. Mother Education Level and Annual Household Income

We found a positive association between maternal education level and PFAS concentrations, suggesting that PFAS sources may derive from different dietary patterns, such as increased consumption of seafood, related nutrition supplements, and environmental conditions [43]. The Aarhus Birth Cohort from Denmark and Mother and Child Cohort study from Norway also found that women with higher education level had higher PFAS levels [24,25]. In contrast, the Taiwan Birth Panel Study revealed a negative association between cord blood PFOS and maternal education level [35]. This may be different sources of exposure in the regions.

However, we also observed that higher annual household income was associated with higher PFOS and PFOA levels. Before the implementation of the PFAS elimination program, PFOS and PFOA with long half-lives played the leading role in the environment, and are still on the market now [5]. Moreover, PFAS mainly derive from products that contain PFOS and PFOA, such as waterproof clothes with higher price, and also the lifestyle pattern and eating habits were also the affecting factors [44,45]. Our results are in agreement with the Maternal-infant Research on Environmental Chemicals in Canada and the Mother and the Child Cohort study from Norway [25,26].

### 4.5. Smoking

Previous studies used self-reported questionnaires to evaluate the smoking status. In our study, we used the cotinine levels at the third trimester to define the maternal smoking status. We found that passive smokers had higher PFOS and PFOA concentrations and active smokers had lower PFUnDA compared to non-smokers. However, one study found no association between PFAS and cotinine levels [36], but cotinine levels were different between passive and active smokers. Kristensen et al. also found no association between PFAS and maternal smoking status, based on self-reports [9]. According to other self-reported studies, Fei et al. found that smokers had lower PFOS and PFOA levels [23]; Brantsaeter et al. found that smokers had lower PFOS levels, but higher PFOA levels [25]. Lauritzen et al. found that smoking status was negatively associated with PFOS levels [37]; Bjerregaard-Olesen et al. also found that smoking status was negatively associated with most PFASs [24]. It is known that cotinine is transmitted to fetus from the mother through the placental and blood flow. Cotinine half-life is 16 h and total clearance occurs in 48 h [46]; however, PFAS half-life is approximately four years. Therefore, the placental and blood flow may play an important role in the regulation of metabolism and absorption between mother and fetus. In addition, mother’s lifestyle and environmental conditions may interfere with PFAS regulation.

### 4.6. Drinking

We observed that mothers with an alcohol consumption history had lower PFDA concentration. However, a few studies had explored the association between PFAS and alcohol consumption. Two studies reported no association [9,25], whereas one study found that women who consumed alcohol before or during pregnancy had higher PFAS levels [24]. The association may be affected by lifestyle or dietary habits or regulated by body fluid and blood circulation [47]. Currently, a few studies had investigated the association between PFAS and alcohol consumption; however, the mechanism is not clear. And most of them found no association or inconsistent association. Whether drinking of alcohol affects excretion of PFAS would be an interesting issue to investigate.

### 4.7. Temporal Trend

In our previous study using a small sample size (*n* = 150), we observed that PFOS and PFOA concentrations decreased, whereas PFNA and PFDA increased [29]. In the present study, we set January 2006 as a cut-off time point and we observed that PFHxS concentration did not change before and after January 2006, whereas PFOS concentration showed a decreasing trend during both periods. PFOA concentration decreased after January 2006. PFNA and PFDA concentrations increased before January 2006, but did not change after that. PFUnDA, PFDoDA, and PFTrDA concentrations increased before January 2006, but decreased after that. To date, most studies reported various levels of PFAS. Consistent with a study conducted during the same period as ours, Eriksson et al. reported that PFHxS, PFOS, and PFOA levels decreased from 2002 to 2013, and PFNA and PFTrDA started decreasing since 2006, in Australia [27]. In other mother-infant related studies conducted in a different time period, Wang et al., 2011 reported that PFOS decreased, whereas PFNA and PFDA increased from the 1960s to 2009; in addition, PFHxS and PFOA started decreasing since the 1980s [7]. Glynn et al. observed that PFHxS, PFNA, and PFDA increased, while PFOS and PFOA decreased between 1996 and 2010 [48]. An increase in PFOS was reported, although no changes over time were observed for PFOA and PFNA by Ode et al. [36]. However, Wilhelm et al. only reported that maternal PFOS levels were higher between 2000–2002 than between 2007–2008; maternal PFNA levels were lower between 2000–2002 than between 2007–2008, suggesting that there was a decreasing trend of PFOS and an increasing trend of PFNA [28]. However, Bjerregaard-Olesen et al. reported that all PFAS, including PFHxS, PFOS, PFOS, PFNA, PFDA, PFUnDA decreased between 2008–2013. Berg et al. reported that PFOS, PFOA, PFHxS, and PFNA levels decreased during the recruitment period of 807 days [6]. Although the sampling time varied for most studies and only one time in the study, we found that PFOS and PFOA levels decreased gradually, suggesting that the international regulation of PFOS and PFOA was successful, especially after 2006; however, there should be more effort regarding PFAS of different carbon chain length. In addition, it is still unclear how people were exposed to PFAS, while blood levels of PFASs were relatively lower in Hokkaido than in other countries [49] and the action would not produce an abrupt end to PFAS exposure. However, PFAS do exist in Japan in the environment [10,50]. We found an association between maternal characteristics and PFAS, but PFAS in the environment should be continuously monitored to understand mother exposure to PFAS in the future.

There were some limitations in our study. Some information regarding pregnant women was based on self-reports, thus there may be a bias for some characteristics, such as for maternal smoking status; however, we used plasma cotinine levels to address this issue. Furthermore, we had no information regarding women’s previous and current dietary, lifestyle, and living habits, which may affect PFAS levels. Finally, we had limited information regarding the background and source of PFAS, which may also influence PFAS levels.

To our knowledge, this study was the first to explore the association between PFAS levels and maternal characteristics during pregnancy, based on a large birth cohort in Asia. Moreover, we measured the levels from 11 types of PFAS in pregnant women, including PFAS with carbon chains longer than eight carbons, which have been reported by only a few studies. We also used plasma cotinine levels to evaluate the maternal smoking status, which was different from previous studies that were based on self-reports.

## 5. Conclusions

We found that PFAS levels varied between different maternal characteristics including maternal delivery age, pre-pregnant BMI, parity, maternal education level, smoking status, alcohol consumption history, and annual household income. PFAS temporal trend changed from 2003 to 2012, except for PFHxS levels. In the future, we should take into account or collect more information regarding the sources of PFAS exposure to investigate causal relationships.

## Figures and Tables

**Table 1:** Maternal basic characteristics (*n* = 2123).

	*n*	%
Age at delivery (years)		
<25	183	8.62
25~29	607	28.58
30~34	882	41.53
≥35	451	21.28
Prepregnancy BMI		
<18.5	385	18.13
18.5~24.9	1554	73.21
≥25	184	8.66
Parity		
0	962	45.29
1	818	38.56
≥2	343	16.15
Education level (years)		
≤9	64	3.01
10~12	879	41.38
13~16	933	43.97
>16	247	11.63
Cotinine Level in 3rd trimester		
Non-smoker (<0.22 ng/mL)	929	43.74
Passive smoker (0.22–11.49 ng/mL)	1017	47.88
Active smoker (>11.49 ng/mL)	177	8.38
Alcohol consumption history		
No	912	42.94
Yes	1211	57.06
Annual housed income (million yen)		
<3	422	19.87
3~5	960	45.20
>5	741	34.93

BMI, body mass index.

**Table 2:** Distribution of 11 PFAS in plasma from pregnant women.

	MDL ^a^	*n*	%	GM	Mean	Min	25th	50th	75th	Max
PFHxS (C6)	0.2	1732	81.54	0.34	0.37	<0.2	0.26	0.33	0.42	3.39
PFHxA (C6)	0.1	976	45.95	0.16	0.16	<0.1	<0.1	<0.1	0.18	0.69
PFHpA (C7)	0.1	740	34.84	0.16	0.18	<0.1	<0.1	<0.1	0.20	1.02
PFOS (C8)	0.3	2123	99.95	4.96	5.74	0.81	3.66	4.96	6.79	30.28
PFOA (C8)	0.2	2123	99.95	2.06	2.64	0.25	1.29	2.00	3.24	24.88
PFNA (C9)	0.3	2121	99.86	1.19	1.35	0.32	0.87	1.15	1.57	13.19
PFDA (C10)	0.1	2110	99.34	0.51	0.57	<0.1	0.39	0.52	0.69	2.43
PFUnDA (C11)	0.1	2117	99.67	1.35	1.50	0.11	1.02	1.40	1.87	5.89
PFDoDA (C12)	0.1	1915	90.16	0.19	0.21	<0.1	0.15	0.19	0.24	0.73
PFTrDA (C13)	0.1	2073	97.60	0.32	0.35	<0.1	0.25	0.33	0.42	1.33
PFTeDA (C14)	0.1	308	14.50	0.12	0.12	<0.1	<0.1	<0.1	<0.1	0.30

PFHxS, prfluorohexane sulfonate; PFHxA, perfluorohexanoic acid; PFHpA, perfluoroheptanoic acid; PFOS, perfluorooctane sulfonate; PFOA, perfluorooctanoic acid; PFNA, perfluorononanoic acid; PFDA, perfluorodecanoic acid; PFUnDA, perfluoroundecanoic acid; PFDoDA, perfluoro-dodecanoic acid; PFTrDA, perfluorotridecanoic acid; PFTeDA, perfluorotetradecanoic acid. ^a^ MDL: method detection limit.

**Table 3:** Mean value and 95% confidence intervals of PFAS concentration in each characteristic on pregnant women ^a^.

	PFHxS	PFOS	PFOA	PFNA	PFDA	PFUnDA	PFDoDA	PFTrDA
Age at delivery (years)								
<25	0.298 (0.276, 0.320)	**5.211 (4.857**, **5.565)**	2.797 (2.515, 3.080)	1.264 (1.184, 1.345)	0.539 (0.503, 0.576)	**1.326 (1.221**, **1.431)**	0.176 (0.164, 0.189)	**0.318 (0.297**, **0.339)**
25~29	0.334 (0.317, 0.350)	**6.080 (5.821**, **6.339)**	2.779 (2.596, 2.962)	1.346 (1.284, 1.408)	0.569 (0.547, 0.592)	**1.476 (1.422**, **1.530)**	0.187 (0.181, 0.194)	**0.338 (0.326**, **0.349)**
30~34	0.320 (0.305, 0.335)	**5.295 (5.136**, **5.455)**	2.588 (2.448, 2.728)	1.368 (1.304, 1.431)	0.560 (0.543, 0.578)	**1.522 (1.475**, **1.568)**	0.193 (0.187, 0.198)	**0.350 (0.340**, **0.361)**
≥35	0.322 (0.304, 0.341)	**5.391 (5.138**, **5.651)**	2.497 (2.263, 2.914)	1.365 (1.277, 1.389)	0.573 (0.546, 0.600)	**1.559 (1.486**, **1.630)**	0.193 (0.185, 0.201)	**0.356 (0.342**, **0.352)**
Prepregnancy BMI								
<18.5	0.327 (0.304, 0.351)	**5.800 (5.512**, **6.088)**	2.665 (2.470, 2.859)	1.436 (1.335, 1.534)	**0.589 (0.559**, **0.617)**	1.559 (1.472, 1.640)	**0.200 (0.191**, **0.209)**	**0.360 (0.343**, **0.378)**
18.5~24.9	0.322 (0.312, 0.332)	**5.532 (5.382**, **5.654)**	2.668 (2.545, 2.774)	1.344 (1.298, 1.383)	**0.564 (0.548**, **0.576)**	1.507 (1.466, 1.535)	**0.191 (0.187**, **0.195)**	**0.347 (0.339**, **0.354)**
≥25	0.317 (0.284, 0.348)	**5.109 (4.689**, **5.518)**	2.402 (2.099, 2.733)	1.268 (1.154, 1.389)	**0.529 (0.497**, **0.563)**	1.373 (1.284, 1.461)	**0.161 (0.151**, **0.171)**	**0.306 (0.285**, **0.327)**
Parity								
0	**0.361 (0.346**, **0.375)**	**6.209 (5.993**, **6.371)**	**3.347 (3.189**, **3.514)**	**1.517 (1.466**, **1.586)**	**0.603 (0.587**, **0.626)**	1.494 (1.450, 1.542)	0.189 (0.185, 0.195)	0.343 (0.333, 0.352)
1	**0.295 (0.280**, **0.309)**	**5.193 (4.992**, **5.347)**	**2.173 (2.039**, **2.289)**	**1.245 (1.183**, **1.301)**	**0.533 (0.516**, **0.550)**	1.506 (1.449, 1.548)	0.191 (0.185, 0.196)	0.349 (0.336, 0.357)
≥2	**0.280 (0.266**, **0.299)**	**4.604 (4.364**, **4.804)**	**1.768 (1.641**, **1.913)**	**1.119 (1.065**, **1.182)**	**0.517 (0.494**, **0.542)**	1.515 (1.439, 1.585)	0.187 (0.178, 0.195)	0.349 (0.333, 0.365)
Education level (years)								
≤9	**0.330 (0.287**, **0.372)**	**4.901 (4.220**, **5.582)**	**2.893 (2.322**, **3.464)**	**1.142 (1.007**, **1.276)**	**0.481 (0.408**, **0.554)**	**1.230 (1.029**, **1.431)**	**0.171 (0.147**, **0.196)**	**0.315 (0.271**, **0.359)**
10~12	**0.301 (0.289**, **0.312)**	**5.332 (5.164**, **5.501)**	**2.502 (2.360**, **2.644)**	**1.268 (1.220**, **1.316)**	**0.542 (0.524**, **0.560)**	**1.437 (1.391**, **1.484)**	**0.184 (0.179**, **0.189)**	**0.336 (0.326**, **0.346)**
13~16	**0.327 (0.313**, **0.341)**	**5.549 (5.366**, **5.736)**	**2.590 (2.453**, **2.718)**	**1.377 (1.314**, **1.437)**	**0.578 (0.560**, **0.596)**	**1.544 (1.497**, **1.590)**	**0.194 (0.188**, **0199)**	**0.354 (0.344**, **0.363)**
>16	**0.381 (0.346**, **0.415)**	**6.349 (5.971**, **6.726)**	**3.263 (2.897**, **3.629)**	**1.613 (1.482**, **1.744)**	**0.612 (0.577**, **0.647)**	**1.626 (1.535**, **1.716)**	**0.200 (0.190**, **0.210)**	**0.355 (0.336**, **0.374)**
Cotinine Level in 3rd trimester								
Non-smoker (<0.22 ng/mL)	0.333 (0.317,.349)	**5.383 (5.207**, **5.560)**	2.568 (2.428, 2.707)	1.377 (1.321, 1.433)	0.568 (0.551, 0.586)	**1.520 (1.475**, **1.566)**	0.193 (0.188, 0.199)	0.344 (0.334, 0.353)
Passive smoker (0.22–11.49 ng/mL)	0.313 (0.302, 0.324)	**5.701 (5.533**, **5.869)**	2.709 (2.567, 2.851)	1.356 (1.299, 1.412)	0.567 (0.550, 0.584)	**1.509 (1.464,.553)**	0.189 (0.183, 0.194)	0.347 (0.338, 0.357)
Active smoker (>11.49 ng/mL)	0.323 (0.293, 0.351)	**5.352 (4.893**, **5.825)**	2.636 (2.312, 2.919)	1.203 (1.107, 1.282)	0.524 (0.482, 0.562)	**1.341 (1.231**, **1.442)**	0.178 (0.165, 0.190)	0.343 (0.318, 0.368)
Alcohol consumption history								
No	0.320 (0.305, 0.334)	5.487 (5.319, 5.655)	2.573 (2.436, 2.711)	1.372 (1.310, 1.433)	0.575 (0.558, 0.593)	1.524 (1.474, 1.573)	0.191 (0.186, 0.197)	0.345 (0.334, 0.355)
Yes	0.325 (0.313, 0.336)	5.567 (5.405, 5.733)	2.692 (2.559, 2.819)	1.338 (1.290, 1.383)	0.555 (0.540, 0.571)	1.482 (1.443, 1.520)	0.189 (0.184, 0.193)	0.346 (0.337, 0.354)
Annual housed income (million yen)								
<3	**0.307 (0.290**, **0.324)**	**5.140 (4.969**, **5.447)**	**2.526 (2.325**, **2.712)**	**1.265 (1.193**, **1.348)**	**0.532 (0.508**, **0.559)**	1.440 (1.371, 1514)	0.186 (0.177, 0.194)	0.342 (0.326, 0.356)
3~5	**0.310 (0.301**, **0.325)**	**5.387 (5.275**, **5.625)**	**2.564 (2.437**, **2.714)**	**1.323 (1.262**, **1.363)**	**0.564 (0.46**, **0.581)**	1.500 (1.445, 1.535)	0.189 (0.183, 0.194)	0.341 (0.330, 0.349)
>5	**0.346 (0.325**, **0.361)**	**5.821 (5.617**, **5.652)**	**2.850 (2.620**, **2.963)**	**1.475 (1.376**, **1.520)**	**0.585 (0.561**, **0.601)**	1.552 (1.493, 1.596)	0.194 (0.188, 0.200)	0.356 (0.344, 0.367)

PFHxS, perfluorohexane sulfonate; PFOS, perfluorooctane sulfonate; PFOA, perfluorooctanoic acid; PFNA, perfluorononanoic acid; PFDA, perfluorodecanoic acid; PFUnDA, perfluoroundecanoic acid; PFDoDA, perfluorododecanoic acid; PFTrDA, perfluorotridecanoic acid; BMI, body mass index; Bold represented *p* value < 0.05; ^a^ one way ANOVA.

**Table 4:** Adjust regression coefficients (β) and 95% confidence intervals in multivariable linear regression model by PFAS in pregnant women.

	PFHxS	PFOS	PFOA	PFNA	PFDA	PFUnDA	PFDoDA	PFTrDA
Age at delivery (years)								
<25	ref	ref	ref	ref	ref	ref	ref	ref
25~29	0.071 (−0.026, 0.169)	**0.114 (0.036**, **0.191)**	−0.028 (−0.136, 0.081)	0.006 (−0.072, 0.085)	0.014 (−0.07, 0.098)	**0.103 (0.015**, **0.191)**	0.046 (−0.04, 0.132)	0.02 (−0.063, 0.104)
30~34	0.028 (−0.068, 0.124)	0.026 (−0.05, 0.103)	−0.049 (−0.156, 0.058)	0.019 (−0.058, 0.097)	0.012 (−0.07, 0.095)	**0.118 (0.031**, **0.205)**	**0.088 (0.003**, **0.173)**	0.063 (−0.02, 0.145)
≥35	0.054 (−0.051, 0.159)	0.064 (−0.02, 0.148)	−0.038 (−0.156, 0.079)	0.041 (−0.044, 0.126)	0.046 (−0.044, 0.136)	**0.131 (0.036**, **0.227)**	0.071 (−0.022, 0.164)	0.078 (−0.012, 0.168)
*p* for trend	0.895	0.574	0.498	0.224	0.272	**0.025**	0.094	**0.026**
Preprgnancy BMI								
<18.5	−0.032 (−0.096, 0.032)	0.034 (−0.018, 0.085)	0.001 (−0.071, 0.072)	**0.058 (0.006**, **0.11)**	0.033 (−0.022, 0.089)	0.035 (−0.023, 0.094)	0.056 (−0.001, 0.113)	0.037 (−0.018, 0.092)
18.5~24.9	ref	ref	ref	ref	ref	ref	ref	ref
≥25	0.021 (−0.067, 0.109)	−0.036 (−0.107, 0.034)	−0.034 (−0.133, 0.064)	−0.012 (−0.083, 0.06)	−0.015 (−0.091, 0.061)	−0.07 (−0.15, 0.009)	**−0.166 (−0.244**, **−0.088)**	**−0.162 (−0.238**, **−0.087)**
Parity								
0	ref	ref	ref	ref	ref	ref	ref	ref
1	**−0.225 (−0.28**, **−0.17)**	**−0.172 (−0.216**, **−0.127)**	**−0.447 (−0.508**, **−0.386)**	**−0.201 (−0.245**, **−0.156)**	**−0.106 (−0.153**, **−0.059)**	0.015 (−0.035, 0.065)	0.006 (−0.043, 0.054)	0.005 (−0.042, 0.052)
≥2	**−0.233 (−0.305**, **−0.16)**	**−0.286 (−0.344**, **−0.227)**	**−0.621 (−0.702**, **−0.54)**	**−0.271 (−0.33**, **−0.212)**	**−0.135 (−0.197**, **−0.072)**	0.025 (−0.042, 0.091)	−0.007 (−0.071, 0.058)	0.021 (−0.042, 0.083)
*p* for trend	**<0.001**	**<0.001**	**<0.001**	**<0.001**	**<0.001**	0.382	0.922	0.582
Education level (years)								
≤9	ref	ref	ref	ref	ref	ref	ref	ref
10~12	−0.145 (−0.291, 0.002)	0.106 (−0.011, 0.224)	−0.173 (−0.337, −0.01)	0.058 (−0.061, 0.176)	**0.159 (0.033**, **0.285)**	**0.212 (0.079**, **0.345)**	0.077 (−0.052, 0.207)	0.067 (−0.059, 0.192)
13~16	−0.087 (−0.234, 0.061)	**0.122 (0.003**, **0.24)**	−0.133 (−0.297, 0.031)	0.099 (−0.021, 0.218)	**0.208 (0.081**, **0.335)**	**0.265 (0.131**, **0.399)**	0.122 (−0.008, 0.253)	0.117 (−0.01, 0.243)
>16	0.016 (−0.145, 0.177)	**0.229 (0.1**, **0.358)**	−0.007 (−0.187, 0.172)	**0.218 (0.088**, **0.349)**	**0.267 (0.128**, **0.406)**	**0.322 (0.176**, **0.468)**	**0.155 (0.013**, **0.298)**	0.106 (−0.032, 0.244)
*p* for trend	**0.004**	**<0.001**	**0.022**	**<0.001**	**<0.001**	**<0.001**	**0.005**	**0.032**
Cotinine Level in 3rd trimester								
Non-smoker (<0.22 ng/mL)	ref	ref	ref	ref	ref	ref	ref	ref
Passive smoker (0.22–11.49 ng/mL)	−0.007 (−0.059, 0.046)	**0.069 (0.027**, **0.111)**	**0.060 (0.002**, **0.119)**	−0.004 (−0.047, 0.038)	−0.002 (−0.047, 0.043)	0.015 (−0.033, 0.062)	−0.01 (−0.057, 0.036)	0.026 (−0.019, 0.071)
Active smoker (>11.49 ng/mL)	0.068 (−0.028, 0.163)	0.034 (−0.042, 0.11)	0.104 (−0.003, 0.210)	−0.043 (−0.121, 0.034)	−0.049 (−0.132, 0.033)	**−0.111 (−0.198**, **−0.025)**	−0.059 (−0.143, 0.026)	0.015 (−0.066, 0.097)
*p* for trend	0.43	**0.019**	**0.021**	0.398	0.404	0.176	0.248	0.381
Alcohol consumption history								
No	ref	ref	ref	ref	ref	ref	ref	ref
Yes	0.012 (−0.013, 0.037)	0.002 (−0.018, 0.022)	0.012 (−0.015, 0.04)	−0.008 (−0.028, 0.012)	**−0.027 (−0.048**, **−0.006)**	−0.019 (−0.041, 0.004)	−0.011 (−0.033, 0.011)	0.001 (−0.021, 0.022)
Annual housed income (million yen)								
<3	ref	ref	ref	ref	ref	ref	ref	ref
3~5	−0.007 (−0.074, 0.059)	0.039 (−0.015, 0.092)	0.015 (−0.059, 0.089)	0.027 (−0.026, 0.081)	0.045 (−0.012, 0.102)	0.019 (−0.041, 0.08)	0.014 (−0.045, 0.072)	−0.016 (−0.073, 0.041)
>5	0.064 (−0.008, 0.136)	**0.094 (0.036**, **0.152)**	**0.111 (0.031**, **0.191)**	**0.08 (0.022**, **0.139)**	0.052 (−0.01, 0.114)	0.034 (−0.031, 0.1)	0.025 (−0.039, 0.089)	0.012 (−0.049, 0.074)
p for trend	**0.041**	**0.001**	**0.003**	**0.004**	0.127	0.304	0.438	0.565

PFHxS, perfluorohexane sulfonate; PFOS, perfluorooctane sulfonate; PFOA, perfluorooctanoic acid; PFNA, perfluorononanoic acid; PFDA, perfluorodecanoic acid; PFUnDA, perfluoroundecanoic acid; PFDoDA, perfluorododecanoic acid; PFTrDA, perfluorotridecanoic acid; BMI, body mass index; Model was adjusted for pre-pregnancy BMI, maternal education, annual housed income, parity, and age at delivery without itself; Bold represented *p* value < 0.05.

## Supplementary Materials

The following are available online at http://www.mdpi.com/1660-4601/15/5/989/s1, Table S1: Crude regression coefficients (ß) and 95% confidence intervals in multivariable linear regression model by PFAS in pregnant women; Table S2: Pregnant women’s blood collection period and sample size at each period; Table S3: Maternal basic characteristics before and after Jan 2006.
