# Supplementary material for: Determinants and Temporal Trends of Perfluoroalkyl Substances in Pregnant Women: The Hokkaido Study on Environment and Children’s Health
Source: Int J Environ Res Public Health. 2018 May 14;15(5):989. doi: 10.3390/ijerph15050989 (PMC5982028; doi:10.3390/ijerph15050989)
Supplement: Supplementary file 1 [file ijerph-15-00989-s001.pdf]

Table S1. Crude regression coefficients (ß) and 95% confidence intervals in multivariable linear regression model by PFAS in pregnant women.

	PFHxS	PFOS	PFOA	PFNA	PFDA	PFUnDA	PFDoDA	PFTrDA
Age at delivery (years)								
<25	ref	ref	ref	ref	ref	ref	ref	ref
25~29	0.087(-0.009,0.184)	**0.128(0.049,0.207)**	0.031(-0.049,0.11)	0.031(-0.049,0.11)	0.043(-0.04,0.126)	**0.144(0.057,0.23)**	0.071(-0.013,0.155)	0.041(-0.041,0.123)
30~34	0.031(-0.062,0.124)	0.021(-0.055,0.097)	0.026(-0.051,0.102)	0.026(-0.051,0.102)	0.035(-0.045,0.114)	**0.167(0.083,0.25)**	**0.112(0.031,0.193)**	**0.083(0.004,0.161)**
>=35	0.034(-0.066,0.135)	0.028(-0.054,0.11)	0.018(-0.064,0.101)	0.02(-0.062,0.103)	0.053(-0.033,0.139)	**0.181(0.091,0.271)**	**0.089(0.002,0.177)**	**0.093(0.009,0.178)**
p for trend	0.559	**0.048**	**<0.001**	0.88	0.383	**0.001**	**0.042**	**0.01**
Preprgnancy BMI								
<18.5	-0.022(-0.088,0.043)	0.047(-0.006,0.1)	0.036(-0.041,0.113)	**0.066(0.012,0.119)**	0.034(-0.022,0.089)	0.023(-0.035,0.082)	0.049(-0.008,0.106)	0.029(-0.026,0.084)
18.5~24.9	ref	ref	ref	ref	ref	ref	ref	ref
>=25	-0.007(-0.096,0.082)	-0.069(-0.141,0.004)	-0.097(-0.203,0.009)	-0.044(-0.117,0.029)	-0.036(-0.113,0.04)	**-0.081(-0.161,-0.001)**	**-0.175(-0.252,-0.097)**	**-0.167(-0.243,-0.092)**
Parity								
0	ref	ref	ref	ref	ref	ref	ref	ref
1	**-0.223(-0.276,-0.169)**	**-0.183(-0.226,-0.14)**	**-0.458(-0.517,-0.398)**	**-0.202(-0.246,-0.159)**	**-0.108(-0.154,-0.062)**	0.015(-0.033,0.064)	0.001(-0.047,0.048)	0.003(-0.043,0.049)
>=2	**-0.226(-0.297,-0.156)**	**-0.298(-0.355,-0.241)**	**-0.631(-0.71,-0.552)**	**-0.27(-0.328,-0.213)**	**-0.136(-0.197,-0.075)**	0.026(-0.038,0.091)	-0.013(-0.076,0.05)	0.019(-0.042,0.08)
p for trend	**<0.001**	**<0.001**	**<0.001**	**<0.001**	**<0.001**	0.382	0.741	0.528
Education level (years)								
<=9	ref	ref	ref	ref	ref	ref	ref	ref
10~12	-0.119(-0.267,0.028)	**0.125(0.004,0.245)**	-0.128(-0.302,0.046)	0.088(-0.032,0.209)	**0.176(0.05,0.302)**	**0.226(0.093,0.358)**	0.088(-0.042,0.217)	0.079(-0.046,0.204)
13~16	-0.047(-0.194,0.101)	**0.16(0.04,0.28)**	-0.057(-0.231,0.117)	**0.152(0.031,0.272)**	**0.239(0.114,0.365)**	**0.292(0.16,0.424)**	**0.146(0.017,0.275)**	**0.142(0.017,0.267)**
>16	0.081(-0.078,0.241)	**0.3(0.17,0.431)**	0.126(-0.062,0.315)	**0.303(0.172,0.434)**	**0.315(0.178,0.451)**	**0.356(0.213,0.5)**	**0.186(0.046,0.327)**	**0.138(0.002,0.273)**
p for trend	**<0.001**	**<0.001**	**<0.001**	**<0.001**	**<0.001**	**<0.001**	**<0.001**	**0.003**
Cotinine Level in 3rd trimester								
Non-smoker (<0.22 ng/mL)	ref	ref	ref	ref	ref	ref	ref	ref
Passive smoker (0.22-11.49	-0.02(-0.072,0.032)	0.059(0.016,0.101)	0.058(-0.003,0.119)	-0.021(-0.064,0.022)	-0.019(-0.063,0.026)	-0.012(-0.059,0.034)	-0.025(-0.071,0.02)	0.008(-0.036,0.052)

ng/ml)								
Active smoker (>11.49 ng/mL)	-0.001(-0.094,0.093)	-0.038(-0.115,0.039)	-0.002(-0.113,0.109)	**-0.123(-0.2,-0.045)**	**-0.108(-0.189,-0.028)**	**-0.164(-0.248,-0.08)**	**-0.094(-0.176,-0.012)**	-0.02(-0.1,0.059)
p for trend	0.658	0.383	0.307	**0.008**	**0.024**	**0.004**	**0.032**	0.893
Alcohol consumption history								
No	ref	ref	ref	ref	ref	ref	ref	ref
Yes	0.012(-0.013,0.037)	0.003(-0.017,0.024)	0.012(-0.017,0.042)	-0.007(-0.028,0.014)	**-0.026(-0.047,-0.004)**	-0.017(-0.039,0.006)	-0.009(-0.031,0.013)	0.002(-0.019,0.023)
Annual housed income (million yen)								
<3	ref	ref	ref	ref	ref	ref	ref	ref
3~5	-0.004(-0.071,0.063)	0.04(-0.014,0.095)	0.006(-0.072,0.085)	0.038(-0.017,0.093)	**0.058(0,0.115)**	0.043(-0.017,0.103)	0.03(-0.028,0.089)	0.001(-0.055,0.058)
>5	**0.081(0.011,0.151)**	**0.107(0.05,0.164)**	**0.107(0.025,0.189)**	**0.112(0.055,0.17)**	**0.086(0.026,0.146)**	**0.086(0.024,0.149)**	0.061(0,0.122)	0.047(-0.012,0.106)
p for trend	**0.008**	**<0.001**	**0.004**	**<0.001**	0.06	0.06	**0.047**	0.076

PFHxS, prfluorohexane sulfonate; PFOS, perfluorooctane sulfonate; PFOA, perfluorooctanoic acid; PFNA, perfluorononanoic acid; PFDA, perfluorodecanoic acid; PFUnDA, perfluoroundecanoic acid; PFDoDA, perfluorododecanoic acid; PFTrDA, perfluorotridecanoic acid; BMI, body mass index; Bold represented p value <0.05.

Table S2. Pregnant women’s blood collection period and sample size at each period.

1	2	3	4	5	6	7	8	9	10	11	12	13	14
2003.8	2004.2	2004.8	2005.2	2005.8	2006.2	2006.8	2007.2	2007.8	2008.2	2008.8	2009.2	2009.8	2010.1
2004.1	2004.7	2005.1	2005.7	**2006.1**	2006.7	2007.1	2007.7	2008.1	2008.7	2009.1	2009.7	2009.12	2012.7
n=92	n=188	n=155	n=154	n=154	n=124	n=166	n=140	n=150	n=161	n=142	n=153	n=256	n=88

Table S3. Maternal basic characteristics before and after Jan 2006.

	before	after	p-value
	N	N	
Age at delivery (years)			
<25	58	125	**0.002**
25~29	258	349	
30~34	297	585	
>=35	130	321	
Preprgnancy BMI			
<18.5	121	264	0.213
18.5~24.9	552	1002	
>=25	70	114	
Parity			
0	326	636	0.127
1	307	511	
>=2	110	233	
Education level (years)			
<=9	26	38	0.266
10~12	324	555	
13~16	314	619	
>16	79	168	
Cotinine Level in 3rd trimester			
Non-smoker (<0.22 ng/mL)	249	680	0.001

Passive smoker (0.22-11.49 ng/mL)	415	602	
Active smoker (>11.49 ng/mL)	79	98	
Alcohol consumption history			
No	320	592	0.940
Yes	423	788	
Annual housed income (million yen)			
<3	136	286	0.385
3~5	339	621	
>5	268	473	

BMI, body mass index.
